# New President of Royal College of Physicians

**Published:** 1918-04-20

**Authors:** 


### NEW PRESIDENT OF ROYAL COLLEGE OF PHYSICIANS.

The College of Physicians has been true to its great traditions in electing Dr. Norman Moore to its presidency. The Fellows of the Royal College of Physicians are the aristocracy of the medical profession, and they form the most democratic body in the world. Unlike the Royal College of Surgeons, whose business is conducted by a Council, which is indeed elected by the Fellows, but which is scarcely responsible to them, for there is no means of calling it to account for any action it may take. The affairs of the College of Physicians are in the hands of the whole body of Fellows, and no action or decision can be taken in the name of the College except the whole of the Fellows in College assembled have had an opportunity of voting upon it. The election of President is effected in the most democratic manner possible. No name is proposed; no name is seconded. Each of the Fellows present writes the name of the man who seems to him most fit for the place, and the Fellow who receives an absolute majority of votes is elected. By no other method could the true and independent opinion of the Fellows be so accurately obtained.

According to the tradition of the College, character, that is to say a reputation for uprightness and honourable dealing, is the first and most important qualification in its President. The qualification next in importance is culture, that is to say, scholarship, learning, and politeness in the old sense of this word, together with the wisdom that should accompany them and result from them. The President of the College of Physicians is in frequent communication with the Government, and with outside bodies, such as the Universities, the Royal Society, and other important institutions, both at home and abroad, and it is necessary that he should be a man who can hold his own with the best of them, both in force of character and in the ornamental qualities of eloquence, personal presence, and dignity. When the College is not in session, the President and the Censor's Board carry on its business, and it is of the greatest importance that the President should be a good man of business, capable of prompt decision and of taking long and wide views. All these qualifications Dr. Norman Moore possesses in a very unusual degree. His personal character is spotless. Few men, either within or without the profession of medicine, can rival him in learning, culture, polish, and urbanity. His eloquence matches anything that even the House of Lords can adduce, and the delivery of his polished periods has a weight and dignity worthy of their matter. He is, withal, an excellent man of business, and has for many years taken a prominent part in various capacities, as Councillor, Censor, and Harveian Librarian, in the business of the College, while in Comitia his suggestions are received with respect, and are almost always adopted. In the long roll of distinguished men who have occupied the Presidential Chair during the four hundred years of the history of the College, including Linacre, Caius, Glisson, Sir Hans Sloane, Sir Henry Halford, Sir Thomas Watson, and Sir William Jenner, there is none more worthy to sustain the dignity of the College than Norman Moore.

